# The Role of Conformational Dynamics and Allostery in the Control of Distinct Efficacies of Agonists to the Glucocorticoid Receptor

**DOI:** 10.3389/fmolb.2022.933676

**Published:** 2022-07-07

**Authors:** Yuxin Shi, Shu Cao, Duan Ni, Jigang Fan, Shaoyong Lu, Mintao Xue

**Affiliations:** ^1^ Department of Pathophysiology, Key Laboratory of Cell Differentiation and Apoptosis of Chinese Ministry of Education, Shanghai Jiao Tong University School of Medicine, Shanghai, China; ^2^ Medicinal Chemistry and Bioinformatics Center, Shanghai Jiao Tong University School of Medicine, Shanghai, China; ^3^ Department of Urology, Ezhou Central Hospital, Hubei, China; ^4^ The Charles Perkins Centre, University of Sydney, Sydney, NSW, Australia; ^5^ Department of Orthopedics, Second Affiliated Hospital of Naval Medical University, Shanghai, China

**Keywords:** glucocorticoid receptor, allosteric communication, allosteric site, molecular dynamics simulation, drug discovery

## Abstract

Glucocorticoid receptor (GR) regulates various cellular functions. Given its broad influence on metabolic activities, it has been the target of drug discovery for decades. However, how drugs induce conformational changes in GR has remained elusive. Herein, we used five GR agonists (dex, AZ938, pred, cor, and dibC) with different efficacies to investigate which aspect of the ligand induced the differences in efficacy. We performed molecular dynamics simulations on the five systems (dex-, AZ938-, pred-, cor-, and dibC-bound systems) and observed a distinct discrepancy in the conformation of the cofactor TIF2. Moreover, we discovered ligand-induced differences regarding the level of conformational changes posed by the binding of cofactor TIF2 and identified a pair of essential residues D590 and T39. We further found a positive correlation between the efficacies of ligands and the interaction of the two binding pockets’ domains, where D590 and T739 were involved, implying their significance in the participation of allosteric communication. Using community network analysis, two essential communities containing D590 and T739 were identified with their connectivity correlating to the efficacy of ligands. The potential communication pathways between these two residues were revealed. These results revealed the underlying mechanism of allosteric communication between the ligand-binding and cofactor-binding pockets and identified a pair of important residues in the allosteric communication pathway, which can serve as a guide for future drug discovery.

## Introduction

**FIGURE 1:**
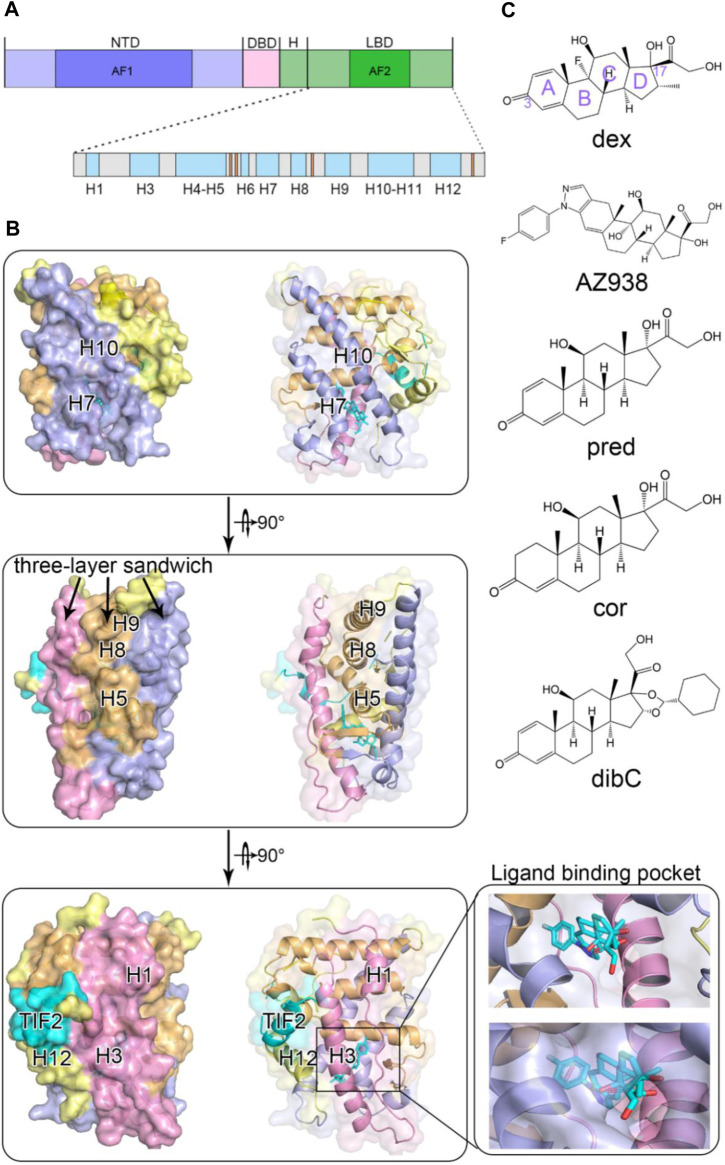
Overall structure of glucocorticoid receptor with agonists and a cofactor. **(A)** domain organization of GR. **(B)** cartoon representation of GR ligand-binding domain with helices colored according to the three-layered sandwich structure. **(C)** chemical structures of five agonists.

Glucocorticoid receptor belongs to the nuclear receptor (NR) superfamily to transduce the signals triggered upon its ligand glucocorticoid (GC) binding (Veleiro et al., 2010; Kadmiel and Cidlowski, 2013; Cain and Cidlowski, 2015). It is broadly implicated in a variety of biological events such as metabolism, proliferation, and apoptosis. Given the critical significance of GR, its structures and related signaling pathways have been intensively investigated in detail. GR comprised three domains, including one N-terminal transactivation domain (NTD), one DNA-binding domain (DBD), and one ligand-binding domain (LBD) (Figure 1A) (Álvarez et al., 2008a). The NTD is intrinsically disordered and contains an activation function 1 (AF-1) transactivation domain, which is responsible for interacting with the coactivator and is responsible for GR’s transcriptional activities. Despite lacking a stable tertiary structure in its intrinsically disordered region (IDR), NTD is essential in the allosteric control of GR’s activity (Li et al., 2017). Li et al*.* (2017) demonstrated that hGR tunes signaling from NTD by producing isoforms differing uniquely in the length of the disordered region. This IDR with a discrepancy in length was believed to propagate structural changes and influence the function of the receptor. On the other hand, the DBD possesses two distinguishable zinc finger regions where DNA anchors. The C-terminal region is where ligands bind, which is also involved in dimerization and interaction with the cofactor through the activation function-2 (AF2) domain (Carson-Jurica et al., 1990; Gronemeyer and Moras, 1995; Kumar and Thompson, 1999; Nagy and Schwabe, 2004). Upon agonists binding, the ligand-dependent AF2 induced conformational changes in GR and accomplished full transactivation function together with AF1 (Goto et al., 2003). The peculiarity of the LBD makes it the most relevant region for the potential interaction of ligand and receptor (Álvarez et al., 2008b).

Due to its critical implication in GR’s functions, LBD structural biology receives considerable research interest. Although intense time has been invested toward this aspect, relatively limited success has been achieved. The first crystal structure of LBD was not successfully obtained until 2002, which formed a complex with its coactivator nuclear receptor coactivator 2 (TIF2) and ligand dexamethasone (Bledsoe et al., 2002). Since then, experimental studies and computational analyses have rapidly accumulated to focus on structural changes of LBD. It is now widely acknowledged that the LBD domain consists of 11 α-helices (H1, H3-H12) and four small β strands (Figure 1A). The protein folds into a canonical three-layer sandwich with a hydrophobic pocket in the shape of a one-side-opened box to accommodate the ligand (Figure 1B). The side of the box consists of three helices (H3, H7, and H11), and H4–H5 forms the top of the box (Edman et al., 2015). The C-terminal AF2 of the receptor has been found to be an important indicator of the ligand’s efficacy. Since it adopts different conformations in distinct agonist-bound GR systems, AF2’s plasticity suggested its contribution to the discrepancy of different agonists’ efficacy (Buttgereit et al., 2018; Köhler et al., 2020; Hu et al., 2022).

GR executed an essential role in cells, bearing the responsibility of both transcriptional activation and non-genomic actions (Jiang et al., 2014; Meijer et al., 2018). In the absence of ligand, GR is predominantly localized in the cytoplasm and bound to either HSP70 or HSP90 and a tyrosine kinase-like c-Src to form a quaternary complex (Weikum et al., 2017; Lee et al., 2021; Karra et al., 2022). When an agonist binds to the GR and alters its structure, it stimulates downstream signaling pathways. The activated GR disassociates from the quaternary complex and moves into the nucleus in the form of homodimers, where it assembles and integrates with glucocorticoid-responsive elements (GREs) (O’Malley and Tsai, 1992; Pratt and Toft, 1997). GREs often sit at the promoters or exons of the target genes, and GR’s binding leads to the recruitment of other factors required for transcription (Jenkins et al., 2001). By regulating different gene expressions, GR manipulates a wide range of cellular activities and thus possesses enormous potential for clinical applications (Darimont et al., 1998; Hu and Lazar, 1999).

GR is emerging as a critical factor for drug discovery especially in carbohydrate, protein, and fat metabolism (Buttgereit, 2020) and immunological disorder-related disease, such as asthma and dermatitis (Cato and Wade, 1996; Köhler et al., 2020). In 1995, there were ∼6.6 million prescriptions relative to GR written in Germany. Until now, ∼10 million drugs are prescribed just for oral corticosteroids each year merely in the United States (Van Staa et al., 2000; Schäcke et al., 2002). Large amounts of efforts have been dedicated over the last several decades by scientists and pharmaceutical companies to enhance the potency of drugs while minimizing side effects by modifying the chemical groups of natural glucocorticoid cortisol (Cain and Cidlowski, 2015). According to a long-standing hypothesis, the adverse effects were induced by dimer-mediated transcriptional activation since the involved genes participate in glucose synthesis and fat metabolism (Meijer et al., 2018). Based on this hypothesis, the goal of drug design is relatively unambiguous, which is to enhance the non-genomic effect and induce GR-protein interaction while impairing the genomic effect of GR-DNA binding (Heck et al., 1994; Reichardt et al., 2001; Meijer et al., 2018). Thitherto, the most common systemic glucocorticoids in clinical treatments are glucocorticoids with good oral bioavailability, which are eliminated mainly by hepatic metabolism and renal excretion of the metabolites. For instance, hydrocortisone (cortisone; cor), prednisolone (pred), methylprednisolone, and dexamethasone (dex) are all commonly used medicines (Thiessen, 1976; Musson et al., 1991). In addition to the traditional drugs on the market, scientists are inventing drugs with more innovative carbon backbones. One of the new compounds is AZ938, a cortivazol analog, which is currently under clinical trial (Styczynski et al., 2005). The chemical structure of AZ938 contains a bulky phenylpyrazole group replacing the C3 ketone of the steroid A ring. Previously, the 3-ketone was thought to be essential as it is conserved among steroid–receptor structures. However, the equivalent activity of cortivazol turned out to be 165-fold higher than prednisolone. Another notable compound is desisobutyryl-ciclesonide (dibC), which is the active metabolite of ciclesonide. It was proved to modulate *in vitro* allergen-driven activation of blood mononuclear cells and allergen-specific T-cell blasts (Czock et al., 2005). Unfortunately, despite the prosperity of drug design, a troublesome setback for drug design is that it is hard to separate the anti-inflammatory efficacy from side effects such as diabetes, muscle wasting, and osteoporosis (Schäcke et al., 2002; Gebhardt et al., 2013), which has become a huge disturbance to many people worldwide. Thus, it is becoming urgent to understand the structural mechanisms of GR–agonist interaction to better optimize drug design (Nussinov and Tsai, 2013). Even so, the underlying mechanism regarding interactions of GR and agonists is still unclear. In addition, the challenge of drug resistance requires an urgent design of new drugs (Fan et al., 2021; Liang et al., 2021). Without accurate comprehension of the relationship between ligands and GR as guidance, it will be difficult to optimize the current drugs and invent new ones with high efficacy and few side effects (Lu et al., 2016; Feng et al., 2021; Lu et al., 2021a). Despite this, most of the studies currently are focusing on the allosteric discrepancy between agonist-bound and antagonist-bound GR systems, while few are focusing on the subtle changes that occurred in different agonist-bound GR systems. To tackle the long-standing setbacks of drug design, a study on the regulation of agonists on the GR is imminently needed (Liu and Nussinov, 2016; Lu et al., 2019c).

Here, we chose five typical GR agonists (dex, AZ938, pred, cor, and dibC) (Figure 1C) with different efficacies to investigate the mechanism underlying ligand−LBD interactions, accounting for different levels of GR function. The efficacies of the five ligands were previously measured using a transactivation reporter gene assay (Köhler et al., 2020). Compared with the highest effect of dex (100%), AZ938 ranked second with 90% of efficacy, which was followed by pred (86%). DibC and cor turned out to be the least effective (77%). Based on these results, we raised the question that what aspect of ligands induced the difference in efficacies. We carried out molecular dynamics (MD) simulations through a multiple microsecond timescale to explore the underlying allosteric effects and conformational dynamics of the LBD. We focused on the two pockets: the ligand-binding pocket and the cofactor-binding pocket, and their allosteric communication induced by different ligand binding to GR (Lu et al., 2019b). By aligning the representative structure of each system, we found different structural ensembles in the cofactor-binding pocket. Further dissection of conformational landscapes showed that induced by different ligands, dynamics in allosteric regulation was found in the response to cofactor TIF2. Moreover, using molecular mechanics Poisson–Boltzmann surface area (MM/PBSA) calculation and distance analysis, we identified crucial residues that displayed preference for a more stable conformation in dex-bound and AZ938-bound systems (Zhang et al., 2019). On the other hand, dynamic cross-correlation matrices (DCCM) calculations also suggested that regions containing crucial residues exhibited significantly increased correlated motions in dex-bound systems compared to other systems. Finally, community network analysis and allosteric pathway analysis were carried out to reveal the potential communication pathways in each system (Ni et al., 2020). Together, this study investigated the allosteric dynamics between the five systems in detail, expounding the mechanism of interactions between agonists and GR. We expect this dynamic model of allostery will prove to be generally adopted in explaining signaling in all the other GR−agonist systems. Ultimately, we hope that this model can be a guide for chemical modification and optimization of drugs and give insights into novel treatments of concomitant drugs (Shen et al., 2016; Lu et al., 2019a; Lu and Zhang, 2019d; Zhang et al., 2022).

## Materials and Methods

### System Preparation

Three co-crystal structures of GR complexed with agonists (dex−GR, PDB ID: 4UDC; cor−GR, PBD ID: 4P6X; and dibC−GR, PBD ID: 4UDD) were selected from the Protein Data Bank (PDB) as initial structures for MD simulations. The mutated residues were mutated back, and the missing residues were added using the Discovery Studio.

### Molecular Docking

Due to the unavailability of co-crystal structures of GR−AZ938 and GR−pred complexes, molecular docking was performed to generate the 3D structure of these two complexes. The chemical structures of AZ938 and pred were built and pre-optimized using the ChemDraw software. The GR−NN7 complex (PBD ID: 4CSJ) and GR−dex complex (PBD ID: 4UDC) were used as templates for AZ938 and pred, respectively. The following docking procedures were accomplished using the Schrödinger program. The unnecessary water molecules beyond 5 Å and other cofactors were deleted from the template structure using the protein preparation module of Schrödinger. The H-bonds were optimized, and the system energy was minimized. The glide module was then used to generate boxes for docking. The target agonists were loaded into the software and processed by the ligPrep module. Finally, molecular docking was conducted using the Ligand Docking module in SP mode. All the above operations were carried out using default settings and parameters. The resulting docking poses were then analyzed with Pymol and Discovery Studio. Additional minimization of 10,000 steps using the steepest descent algorithm was performed by Discovery Studio to optimize the docking interface.

### MD Simulations

MD simulations were performed on five systems (GR−dex, GR−AZ938, GR−pred, GR−cor, and GR−dibC) using the AMBER18 software (Jang et al., 2020; Li et al., 2020). First, we used Antechamber to create inpcrd and prmtop files for each agonist. Antechamber is a forcefield specifically designed to cover most pharmaceutical molecules and has excellent compatibility with the traditional AMBER forcefield. We loaded the ligand input PDB files and ran the *reduce* to add all the hydrogen to the systems. Then, we transformed the PDB files into Tripos Mol2 format. The AM1-BCC charge model was used to calculate the atomic charges. Utility *parmchk* was applied to create parameter files that can be loaded into LEaP. After loading the parameter files, we ran the LEaP and finally obtained the inpcrd and prmtop files (Bayly et al., 1993; Jakalian et al., 2000; Wang et al., 2004). Second, we obtained all the parameter files of the protein using ff14SB forcefield (Maier et al., 2015) and general Amber forcefield (GAFF). We added hydrogen to all the systems and created a truncated octahedron transferable intermolecular potential three-point (TIP3P) water box (Jorgensen et al., 1983) to approach the environment in physical conditions. We also added Na^+^ and Cl^−^ atoms to neutralize the charge. After the preparation, we operated a protocol using four steps. We operated the minimization step two times. All the atoms in the complex were restrained at 500 kcal mol^−1^Å^−2^ using the steepest descent algorithms at the first time. Other ions and water molecules were minimized within 50,000 cycles (25,000 each for steepest descent and conjugate gradient cycles). At the second time, the systems underwent 50,000 cycles of steepest descent and conjugate gradient minimization each free of restrictions. Then, we heated up the system from 300 ps to 300 K in a canonical ensemble (NVT) with a 700 ps equilibration step. Finally, a 1000 ns MD simulation was carried out in each system with random velocities in isothermal isobaric conditions (NPT) with periodic boundaries. The system was regulated by Langevin dynamics (Uberuaga et al., 2004; Sindhikara et al., 2009) with the collision frequency γ = 1.0. The random seeds were defined by the current time and date. The particle-mesh Ewald (PME) procedure was applied to the long-range electrostatic interaction. A cutoff of 10 Å was set for van der Waals interactions and short-range electrostatics. The SHAKE algorithm was used for the bond’s interaction omitting the H-bonds. Every 5,000 steps, the coordinates would be written into the mdcrd file. The simulation was repeated three times for each complex.

### Cluster Analysis

Cluster analysis was applied to MD trajectories to classify and make sense of information in trajectories. We used the k-means algorithm (Shao et al., 2007), which generated seed points at the start. Then, we iterated all the data points and assigned each of them to the closest seed point. Then, the most representative structures were generated in each cluster for further analysis.

### Molecular Mechanics Poisson–Boltzmann Surface Area (MM/PBSA) Calculations

MM/PBSA was performed using the MMPBSA.py to evaluate the most essential residues in the complex between ligands and the receptor or the cofactors and the receptor with a large contribution to the free binding energy (Chong et al., 2009). The binding free energy was calculated as the total Gibbs free energy changes before and after the binding of ligands or cofactors.

$$\Delta G_{binding}=\Delta G_{complex}-\Delta G_{receptor}-\Delta G_{ligand}.$$

Gibbs free energy mainly consists of three parts: solvation energy (G_solv_), molecular mechanical energy (E_MM_), and the entropic compartments ( −TS ).

$$\begin{array}{l} \Delta G_{binding}\;=\left(E_{MM,\;complex}\;–\;E_{MM,\;ligand}\;-E_{mm,\;receptor}\right)\\ +\;\left(G_{solv,\;complex}\;–\;G_{solv,\;receptor}\;–\;G_{solv,\;ligand\;}\right)\;\\ -\;\left(\;TS_{complex}\;–\;TS_{ligand}\;-\;TS_{receptor}\right). \end{array}$$

Thus, the equation can turn into this formation:

$$\Delta G_{binding}=\Delta E_{MM}\;+\Delta G_{solv}\;–\;TS.$$

Furthermore, $\Delta E_{MM}$ can be divided as follows:

$$\Delta E_{MM}\;=\Delta E_{vdw}\;+\Delta E_{ele}\;+\Delta E_{int},$$

where $\Delta E_{vdw}$ is the van der Waals component, $\Delta E_{ele}$ is the electrostatic component, and $\Delta E_{int}$ is the internal component with angles, bonds, and torsional energies.

According to Poisson–Boltzmann continuum solvent model, $\Delta G_{solv}$ can be divided as:

$$\Delta G_{solv}\;\;=\;\Delta E_{PB}\;+\;\Delta E_{nonpolar},$$

where $\Delta E_{PB}$ stands for the polar part and $\Delta E_{nonpolar}$ stands for the nonpolar part using solvent-accessible surface area (SASA) for calculation.

$$\Delta E_{nonpolar}\;=\gamma SASA\;+\;b.$$

The surface tension parameter was set to 0.00542 $\;kcal\cdot mol^{-1}\cdot {Å}^{-2}$ and the solvent parameter was 0.92 kcal/mol . Given that the five systems were similar with low RMSDs, the −TS could be ignored in our calculations.

### Dynamic Cross-Correlation Matrix (DCCM) Analysis

All trajectories were simplified using only the Cα atoms that were rotated and translated using a least-square fitting procedure (Hünenberger et al., 1995; Li et al., 2021). For the two Cα atoms i and j at time t , the position vectors are $r_{i}\left(t\right)\;$ and $r_{j}\left(t\right)$ , respectively. Correspondingly, the covariance matrix element $c_{ij}$ had the following equation:

$$\begin{array}{l} C_{\mathrm{ij}}=<\left(r_{i}\;-<r_{i}>\right)\;\left(r_{j}\;-\;<r_{j}>\right)>=\;<r_{i}\;r_{j}>\;-\;<r_{i}><r_{j}>\\ =\frac{\Delta t}{t_{aver}}\left[\overset{t_{aver}-\Delta t}{\underset{t=0}{\sum }}r_{i}\left(t\right)r_{j}\left(t\right)-\;\;\frac{\Delta t}{t_{aver}}\left(\overset{t_{aver}-\Delta t}{\underset{t=0}{\sum }}r_{i}\left(t\right))\times (\overset{t_{aver}-\Delta t}{\underset{t=0}{\sum }}r_{j}\left(t\right)\right)\right] \end{array},$$

where Δt stands for the time interval between two frames and $t_{aver}$ stands for average time. Covariance can be used in estimating systems’ entropy (Karplus and Kushick, 1981; Swegat et al., 2003). The cross-correlation matrix element, $c_{ij}$ , was defined as:

$$C_{ij}=\;\frac{c_{ij}}{c_{ii}^{\frac{1}{2}}c_{jj}^{\frac{1}{2}}}=\;\frac{<r_{i}r_{j}>-<r_{i}><r_{j}>}{{\left[\left(<r_{i}^{2}>-<r_{i}{>}^{2}\right)\left(<r_{j}^{2}>-<r_{j}{>}^{2}\right)\right]}^{\frac{1}{2}}}.$$

### Dynamic Network Analysis

In order to reveal the underlying mechanisms of residue–residue interactions, we performed dynamic network analysis to calculate group constitution within the GR. According to this algorithm, the whole GR could be seen as a bunch of nodes. Nodes sitting within a threshold of 4.5Å for at least 75% throughout the trajectories could be seen as a group. We used $d_{ij}=\;-\text{log}\left(|c_{i,j}|\right)$ to calculate the edges between each group. The i and j represented two nodes and $C_{ij}$ could be calculated using the equation mentioned earlier. We also investigated the optimal and suboptimal pathways between two certain nodes using the Floyd–Warshall algorithm. All the procedures could be done using the NetworkView plugin in VMD (Hünenberger et al., 1995; Sethi et al., 2009).

## Results

### Different Agonists’ Binding Induces Distinct TIF2 Conformations

Three independent rounds of 1 μs MD simulations for five systems were conducted to probe into the dynamic conformational changes induced by different agonists. The root-mean-square deviation (RMSD) of the C_α_ atoms was calculated relative to the initial structure to compare the overall conformational dynamics of the five systems. As shown in Figure 1A, all systems reached equilibrium after simulations. The RMSD fell into the range of 2.5–3 Å. Systems possessing ligands with higher efficacy had a slightly lower RMSD, suggesting that different ligands had induced subtle differences in the response of GR. This may indicate that the allosteric effects of ligands might differentially influence the overall energy landscape of GR. To uncover the domain-specific dynamics of GR, we calculated per-residue root mean square fluctuation (RMSF) of each system (Figure 1B). No significant domain-specific conformational differences between the five systems were observed during the simulations.

**FIGURE 2:**
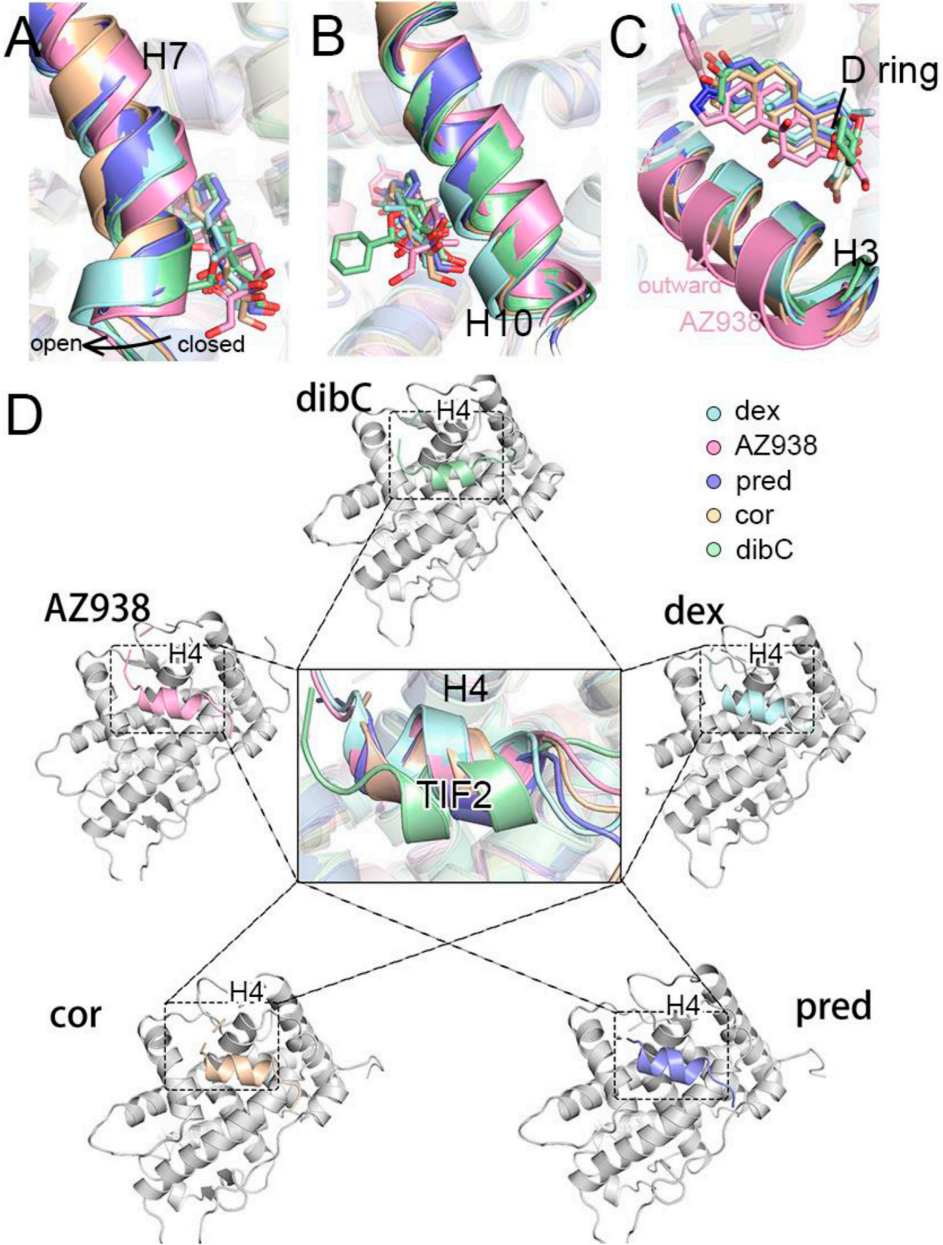
Representative structures of five systems. **(A)** cartoon representations of H7 and ligands. **(B)** cartoon representations of H10 and ligands. **(C)** cartoon representations of H3 and ligands. **(D)** cartoon representations of TIF2 in the cofactor pocket.

To identify the potential region that could contribute to conformational dynamics, we extracted representative structures for each system using cluster analysis. As shown in Figure 2, the representative structure of each system was superimposed on the dex-bound GR with no large structural deviation observed. However, in the systems with less bulky ligands (Figure 1C), the H7 regions formed a slightly “closed” conformation on the ligand-binding site (Figure 2A). In contrast, in the systems with bulky agonists such as dibC and dex, this region formed a more “open” conformation due to the steric hindrance of the bulky chemical groups at the tail of the D-ring. However, the conformation of the H10 appeared to be the opposite. The systems with a more “open” conformation at the H7 tended to be more “closed” at the H10, indicating that the distance between the C terminus of H10 and agonists exhibited a negative correlation with the distance between H7 and agonists (Figure 2B). Exceptionally, AZ938 lacks a conserved 3-ketone head but has a much bigger and electronegative fluoro-phenylpyrazole (Figure 1C). This unique structure of AZ938 resulted in the expansion of the top half of the ligand-binding pocket (Figure 2C). This expansion led to the outward movement of the H3 in the AZ938-bound GR (Figure 2C). More intriguingly, a much stronger correlation was found in the cofactor-binding pocket. By aligning all the representative structures, the combination direction of the cofactor TIF2 was found to be correlated with the efficacies of agonists. As shown in Figure 2D, the TIF2 in the dex-bound GR adopted a conformation closest to the H4. The TIF2 moved in an anticlockwise direction slowly in the sequence of the descending order of efficacy, which pulled the cofactor further away from the H4.

**FIGURE 3:**
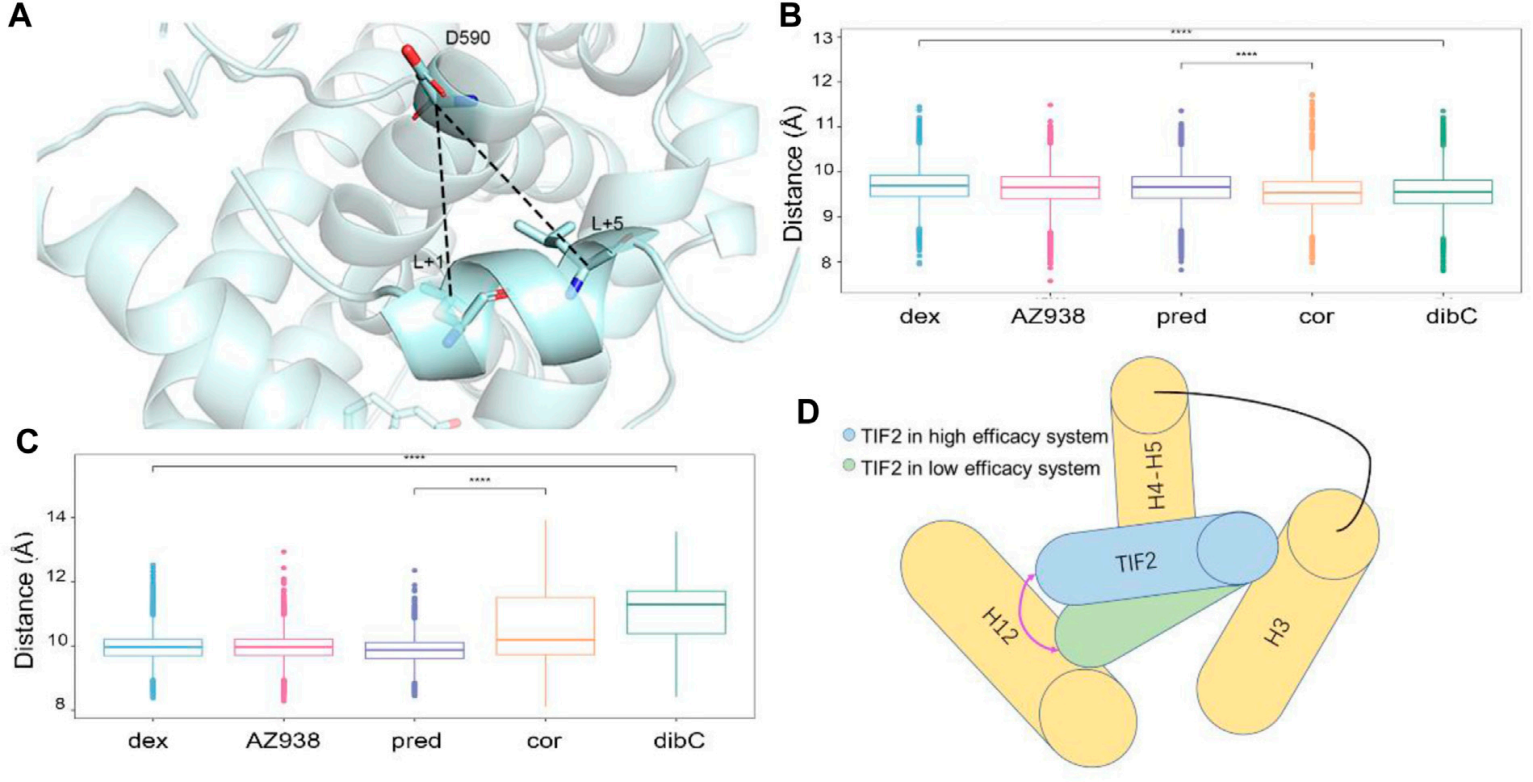
Measurement of the direction of TIF2. **(A)** cartoon structure of the distance measured. Pivotal inter-residue distance reflecting the direction of TIF2 between **(B)** D590 and L+5 and **(C)** D590 and L+1. **(D)** model of TIF2-binding dynamics.

To verify whether this discrepancy in the direction of TIF2 was observed in all three independent replicas of simulations, we measured two pair-wise distances throughout the trajectories (Figure 3A). The distributions of distances between D590 and L+5 indicated that the structure of the C-terminal TIF2 was conserved among the five systems (Figure 3B). However, a distinct discrepancy could be found in the distance between D590 and L+1 (Figure 3C), indicating that within TIF2, the N terminus was dynamic while the C terminus was relatively stable. The distances between D590 and L+1 were roughly consistent with the order of efficacy, implying a significant role of D590 in the communication with the cofactor TIF2. In addition, no significance was found for distances between dex- and AZ938-bound systems, elucidating that the adopted conformation of these two was preferential for higher efficacy (Figure 3B). Interestingly, the fluctuation of distances in the dibC- and cor-bound systems was much larger than that in the rest of the systems, suggesting that they went through severe vibration during the simulation, which illustrated that interaction with D590 could also be important in the stability of TIF2. Previous studies had already investigated the important residues in the cofactor-binding pocket, which interacted with the conserved sequence (LXXLL) on the TIF2 (Necela and Cidlowski, 2003; Liu X. et al., 2019). The D590 on the H4 was proved to be one of the essential residues. This gave us insights into the importance of D590. Thus, we hypothesized that D590 could be an essential residue in the change of the TIF2 conformation.

### Communication Between Ligand- and Cofactor-Binding Pockets Indicates Connection of the Regulation Between Two Pockets to Efficacy Discrepancy

**FIGURE 4:**
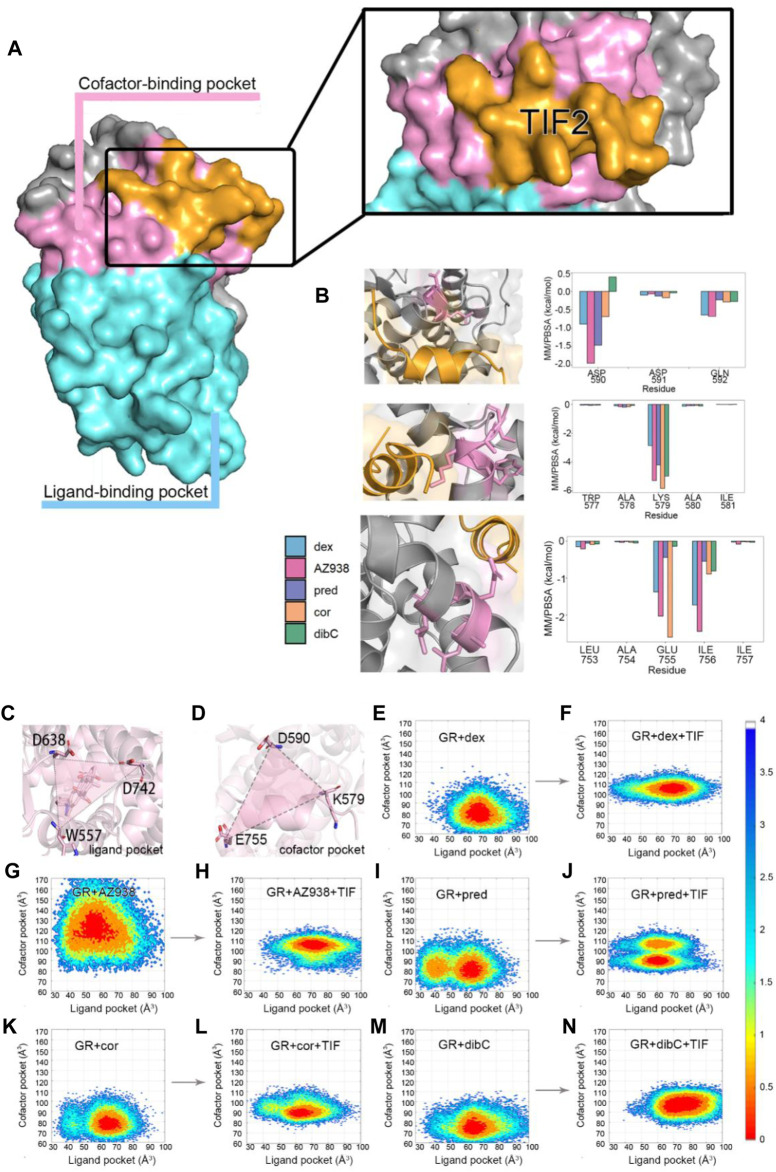
**(A)** cartoon representation of two pockets. **(B)** MM/PBSA of three helices with crucial residues. **(C)** cartoon representation of parameter representing ligand pocket. **(D)** cartoon representation of parameter representing cofactor pocket. Conformation FEL of dex, AZ938, pred, cor, and dibC with or without TIF2 binding **(E**–**N)**. The landscape was generated with Δ_*D638-D742-W557*_ and Δ_*D590-K579-E755*_.

The superposition of representative structures had important implications for allosteric communication in the GR. Consequently, the free energy landscape was projected onto the 2D space using parameters reflecting the situations of pockets (Figure 4) (Lu et al., 2021b). To quantify the influence of residues in the binding pockets on the energetics of TIF2 binding, molecular mechanics Poisson–Boltzmann surface area (MM/PBSA) was employed to compute the binding free energy (ΔG_*binding*_) of TIF2 to GR, which was divided among each residue (Table 1). The lower binding free energy indicated a stronger interaction between TIF2 and the residue. In each of the three helices that surrounded the cofactor-binding pocket, we selected three residues with a large contribution to MM/PBSA. D590, K579, and E755 were selected, respectively, which was consistent with the results in previous studies (Suino-Powell et al., 2008; Veleiro et al., 2010; Alves et al., 2020) to mimic the area of the cofactor-binding pocket (Figure 4A).

**TABLE 1:** Free energy analysis (kcal/mol) for K579, D590, and E755.

K579^a^	Dex-bound system	AZ938-bound system	Pred-bound system	Cor-bound system	DibC-bound system
ΔE*vdw*	−2.21 (0.93)	−2.26 (1.13)	−2.06 (0.97)	−1.53 (1.02)	−1.56 (1.17)
ΔE*ele*	−88.93 (7.53)	−101.14 (9.71)	−85.06 (9.44)	−93.99 (5.62)	−101.54 (7.41)
ΔE*nonpolar*	−0.62 (0.08)	−0.66 (0.06)	−0.63 (0.08)	−0.71 (0.07)	−0.65 (0.06)
ΔE*solv*	88.90 (6.76)	98.77 (7.66)	83.55 (7.97)	90.40 (4.49)	98.76 (6.20)
ΔE*binding*	−2.86 (1.31)	−5.29 (2.06)	−4.20 (1.75)	−5.83 (1.41)	−4.98 (1.56)
**D590**	**Dex-bound system**	**AZ938-bound system**	**Pred-bound system**	**Cor-bound system**	**DibC-bound system**
ΔE*vdw*	−0.09 (0.52)	0.17 (0.87)	0.13 (0.89)	−0.36 (0.61)	−0.43 (0.20)
ΔE*ele*	−3.50 (10.86)	−8.11 (3.49)	−23.41 (10.31)	−27.30 (8.66)	10.41 (4.57)
ΔE*nonpolar*	−0.08 (0.06)	−0.16 (0.02)	−0.09 (0.06)	−0.15 (0.06)	−0.05 (0.05)
ΔE*solv*	2.79 (9.43)	6.16 (3.18)	21.92 (8.57)	27.14 (8.35)	−9.54 (4.56)
ΔE*binding*	−0.89 (1.37)	−1.95 (0.76)	−1.46 (1.75)	−0.68 (0.71)	0.39 (0.32)
**E755**	**Dex-bound system**	**AZ938-bound system**	**Pred-bound system**	**Cor-bound system**	**DibC-bound system**
ΔEvdw	−2.33 (0.73)	−2.54 (0.97)	−1.12 (0.68)	−2.40 (0.78)	−1.46 (0.88)
ΔE*ele*	−20.95 (5.65)	−32.31 (3.29)	−44.89 (11.18)	−62.71 (5.55)	−24.10 (3.85)
ΔE*nonpolar*	−0.51 (0.07)	−0.57 (0.03)	−0.40 (0.06)	−0.54 (0.06)	−0.41 (0.08)
ΔE*solv*	22.40 (5.33)	33.38 (3.07)	45.97 (10.61)	63.05 (5.44)	25.82 (3.94)
ΔE*binding*	−1.37 (1.35)	−2.03 (1.22)	−0.44 (1.37)	−2.60 (1.02)	−0.14 (0.82)

a Numbers in the parentheses are the standard deviations.

Consequently, D590, K579, and E755 were chosen to be the three residues defining the parameter of the triangle that reflected the relative degree of openness of cofactor-binding pockets (Figure 4B). The other parameter reflecting the openness of the ligand-binding pocket was defined by the triangle representing the ligand-binding pocket, which was formed by three residues (W557, D638, and D742) shown in Figure 4C. They were all located at the terminus of the helices constituting the ligand-binding pocket, which reflected the fluctuation of the pocket sensitively. The two areas of triangles were used as the parameters to generate the two-dimensional landscape for each system, which reflected the correlation of the openness of the two pockets. Additional five systems without the cofactor TIF2 have also conducted simulations for the purpose of comparing the landscape before and after the binding of the cofactor. The same parameters were used for the two-dimensional landscape for the five systems without TIF2. By comparing the distribution of the area of the two pockets, we could profile the difference of each system in the response to TIF2’s binding.

As shown in Figure 4, two distinct states were observed before and after the binding of TIF2. Before the binding of the cofactor, the five systems mutually exhibited a conformational state with the ligand-binding pocket area of approximately 65 Å^2^. The area of the cofactor-binding pocket was around 80 Å^2^ in the mutual state with AZ938 to be an exception. A trend for a second preferential conformation at the left of the original one was also discovered in the pred-, cor-, and dibC-bound systems. After cofactor binding, the parameters condensed into a state at the up-right of the plot, with both parameters enlarged. The binding of TIF2 not only influenced the area of the cofactor-binding pocket but also affected the ligand-binding pocket, which implied the allosteric communication between the ligand-binding pocket and cofactor-binding pocket. Despite having inconsistent landscapes before the binding of TIF2, all systems converged their conformational landscape after TIF2’s combination. Intriguingly, parameter Δ_*D590-K579-E755*_ in the dex- and AZ938-bound systems increased significantly to around 100–110 Å (C^up^), which was 10 Å more than the increase in the cor-bound and dibC-bound systems (C^down^). This illustrated that the level of conformational changes induced by the binding of TIF2 was different in each system, probably by influencing the interaction between the two pockets, which might result in the different efficacies of agonists. Interestingly, the coexistence of C^up^ and C^down^ was observed in the pred-bound system, which exhibited features of both agonists with high efficacy (dex-bound and AZ938-bound systems) and low efficacy (cor-bound and dibC-bound systems). These results further verified that these two features regarding the area of two pockets may sensitively reflect the efficacy of the agonists. After the binding of TIF2, the constriction of the ligand-binding pocket conformation was much stronger in the pred-bound system than that in the dibC- and cor-bound systems, implying a stronger response toward the binding of the cofactor in the pred-bound system. Altogether, the results indicated the pocket conformational changes induced by the TIF2 could reflect the efficacies of ligands.

### Representative Structures Indicate That D590 May Be an Important Residue

**FIGURE 5:**
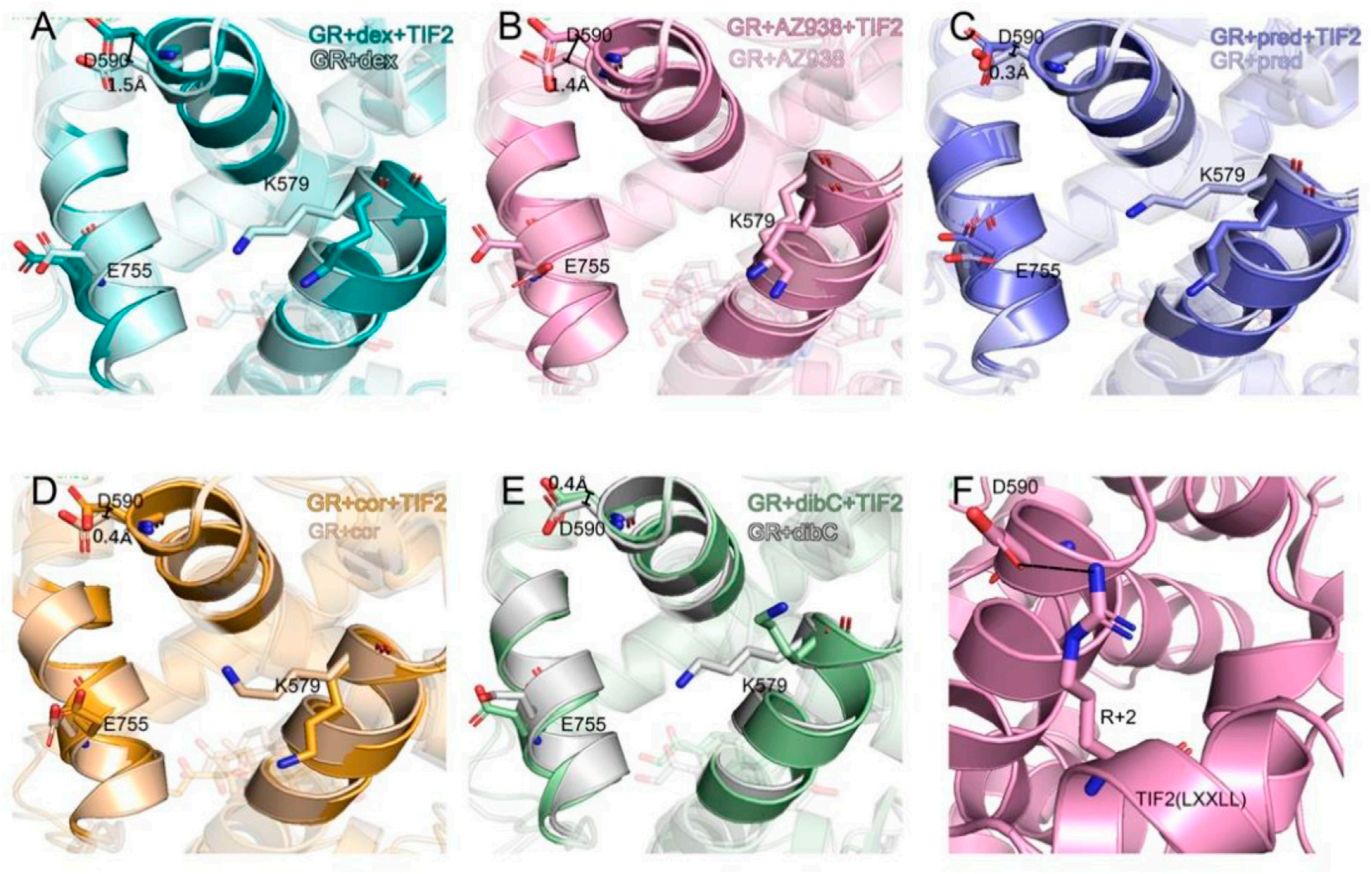
Representative structures of the dex-bound system **(A)**, AZ938-bound system **(B)**, pred-bound system **(C)**, cor-bound system **(D)**, and dibC-bound system **(E)**. In each system, TIF2-bound and TIF2-unbound systems were aligned. Hydrogen bonds between D590 and R+2 are shown in **(F)**. The distance of CB atom before and after the binding of TIF2 was determined.

To further investigate the conformation of the chosen residues in the cofactor pocket, the representative structures were extracted from each two-dimensional landscape (Figure 5). Obvious expansion of the three helices forming the cofactor-binding pocket (H3, H4, and H12) occurred in the dex- and AZ938-bound systems (Figures 5A, B). In the cor- and dibC-bound systems, no significant expansion was observed (Figures 5D, E). The outward movement of the H3 that contains K579 was the most distinct one among the three helices. After the binding of TIF2, K579 all rotated outward, except the one in the dibC-bound system, which flipped away and formed a weak interaction with TIF2. The expansion of H4 only occurred in dex-bound and AZ938-bound systems. In the systems of TIF2-bound and TIF2-unbound, no significant changes occurred in the representative structures of H4 in pred-bound, cor-bound, and dibC-bound systems, which only underwent slight rotation in D590. However, obvious outward movements of D590 and H4 were observed in dex and AZ938. The dynamic conformation of D590 induced a strong interaction between the O atoms in D590 and H atoms in the conserved sequence of LXXLL (Figure 5F), which participated in the stabilization of TIF2. The LXXLL was important in the binding of the AF2 and activation of transcription, thereby having direct relationships with agonists’ efficacy (Heery et al., 1997; Torchia et al., 1997; Plevin et al., 2005). The unique conformational dynamics in dex-bound and AZ938-bound systems implied an important conformation contributing to the higher efficacy of dex and AZ938 (Heery et al., 1997).

**FIGURE 6:**
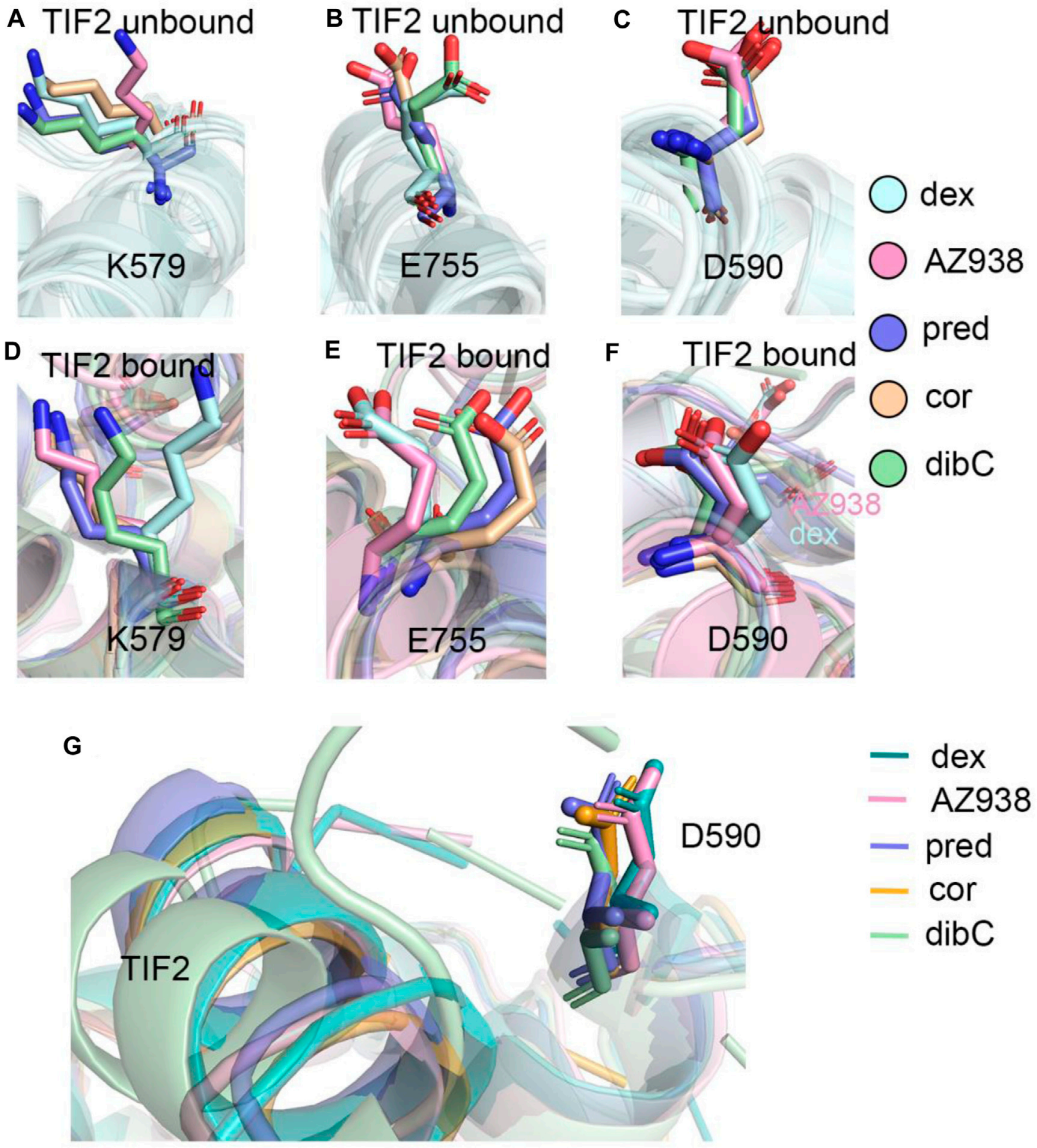
**(A)** representative structures of K579 in the TIF2-unbound systems. **(B)** representative structures of E755 in the TIF2-unbound systems. **(C)** representative structures of D590 in the TIF2-unbound systems. **(D)** representative structures of K579 in TIF2-bound systems. **(E)** representative structure of E755 in TIF2-bound systems. **(F)** representative structure of D590 in TIF2-bound systems. **(G)** representative structure of TIF2 and D590 in TIF2-bound systems.

Given the unique expansion of D590 in dex-bound and AZ938-bound systems, the D590 was further investigated given that it might be a crucial residue in the allosteric communication between the ligand-binding pocket and cofactor-binding pocket. K579 and E755 were observed to have various conformations before and after the binding of TIF2 (Figure 6). No evidence showed that the pattern of K579 and E755 conformation had a relation with the order of efficacy between different systems (Figures 6A, B). However, the conformation of D590 was consistent among the five systems both before and after the binding of TIF2, respectively (Figures 6C–F). The conformation of D590 almost overlapped in the five systems of TIF-unbonded GR. However, a discrepancy was shown in Figure 6F after the binding of TIF2, with the D590 in dex and AZ938 moved slightly outward and separated from the rest of D590 in other systems, despite the overall conformation being consistent between the five systems. This was accompanied by the tight loading of TIF2, which pushed the D590 away from the original conformation (Figure 6G), suggesting an underlying mechanism that TIF2’s binding may be related to the conformational dynamics of D590. Altogether, the representative structures between the 10 systems revealed a potential important residue for communications between the ligand-binding pocket and cofactor-binding pocket.

### Identification of Two Important Residues in the Ligand-Binding Pocket and Cofactor-Binding Pocket

**FIGURE 7:**
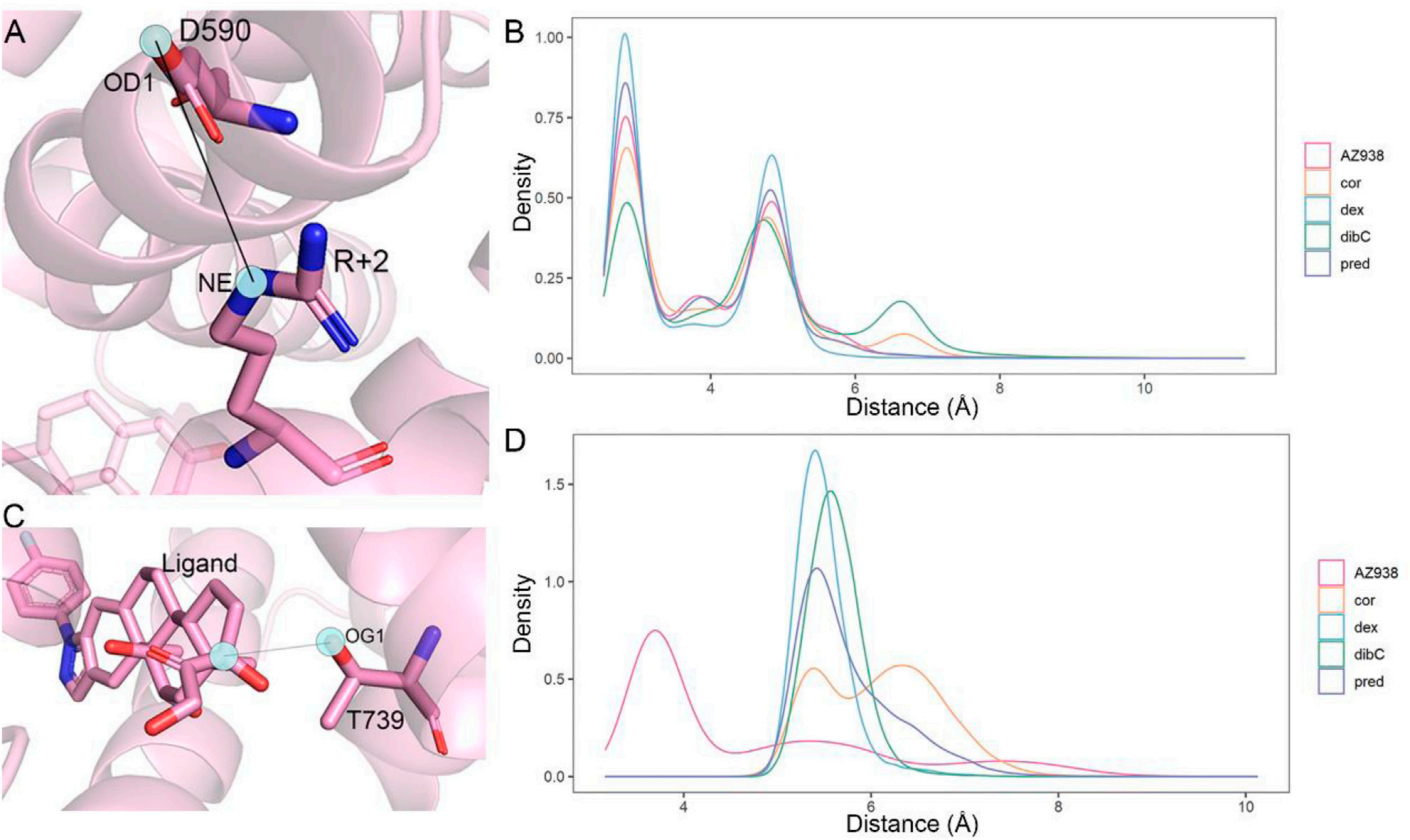
Distance measurement of two pairwise atoms. **(A)** cartoon representation of the distance between OD1 in D590 and R+2 in NE. **(B)** distance distribution density of D1 in D590 and NE in R+2 in five systems. **(C)** cartoon representation of the distance between OG1 in T739 and the C atom in agonists. **(D)** distance distribution density of OG1 in T739 and corresponding C atoms in each agonist (C25 in the AZ938-bound system, C15 in the dibC-bound system, C18 in the pred-bound system, and C17 in dex- and cor-bound systems).

Comparative analyses of the representative structure of the cofactor-binding pocket emphasized the importance of the special relationship between D590 and TIF2. Previous crystal structure analysis also revealed that D590 formed vital hydrogen bonds with the conserved residue R+2 on TIF2 (Suino-Powell et al., 2008). Thus, we calculated the distance distribution of D590 and R+2. Since the oxygen atoms in D590 could form various hydrogen bonds with different N atoms in R+2, we analyzed one pair that could best represent the relationship between these two residues. As shown in Figure 7A, the atom OD1 and atom NE were selected from D590 and R+2, respectively, for distance measurement since these two atoms formed a stable hydrogen bond throughout the three replicas of simulations. The density distribution of distances was shown in Figure 7B. Dex-bound system, the system with the most obvious expansion of D590, had the highest peak of density distribution within 5 Å, while the dibC-bound system had the lowest distribution of distances in this region. The distribution of the dex-bound system rapidly fell to zero beyond 3.5 Å of the distance. The distribution peak of other systems was also significantly lower than that of the dex-bound system. This indicated that the dex-bound system was the most likely system to form the hydrogen bonds between OD1 in D590 and NE in R+2 since hydrogen bonds were considered unable to form in two atoms with a distance larger than 3.5 Å. The highest peaks of the dex-bound system might correspond to the preferential structures of hydrogen bonds, which persistently existed during simulations. Intriguingly, another small peak at a distance of around 7 Å was also observed in dibC-bound and cor-bound systems, where the hydrogen bonds were almost unlikely to form. This implied that dibC-bound and cor-bound systems had an additional sub-preferential conformation in a state that D590 would not form hydrogen bonds with R+2. All these properties of the density distribution illustrated that the dex-bound system might be the most suitable for the formation of hydrogen bonds between OD1 in D590 and NE in R+2, while cor- and dibC-bound systems were less favorable for the formation of hydrogen bonds. The conserved hydrogen bond served as a connection between the GR and TIF2 and was thought to have contributed to the efficacy of ligands. Therefore, this finding agreed to the efficacy order of the five systems.

We next attempt to investigate other residues within the ligand-binding pocket which might also be important for allosteric communication between two pockets. MM/PBSA analysis for the binding of ligand to the GR was carried out, and the result was further decomposed into every residue forming the ligand-binding pocket. Consistent with previous studies, T739 was identified as one of the most important residues for the binding of ligands (Table 2). Among the five systems, the T739 had a consistently large contribution to the binding free energy of ligand to the GR. Thus, we measured the distance between the T739 and the ligands in our five simulation systems, respectively, which was considered an important interaction between the ligand and its pocket. Given that different ligands had distinct structures and tended to have distinct preferences for oxygen atom to form the hydrogen bond with the T739, we selected a conserved atom that was related to all the oxygen possible in forming the hydrogen bond with the T739 (C25 in the AZ938-bound system shown in Figure 7C, C15 in the dibC-bound system, and C17 in dex-, pred-, and cor-bound systems). As shown in Figure 7D, the dex-bound system had the highest density distribution of the distance at around 5 Å. The crest of the pred-bound system was slightly farther than the dex-bound system located at around 4.5 Å. The cor-bound system presented two crests, both of which were farther than 5 Å, indicating a less favorable condition to form hydrogen bonds between the ligand and T739. AZ938 was an exception with an obvious smaller distance between the ligand and T739. This was due to the six rings of AZ938 which contributed to the elongated chemical structure. In order to fit into the ligand-binding pocket with this unusual structure, AZ938 folded its tail at the D ring toward the direction of H7, while the C25 in AZ938 was exposed to the T739. As a result, the distance distribution of the AZ938-bound system contributed to the decrease in the peak distance. However, from the position of the peaks in the five systems, we concluded that the distance between the T739 and the selected atom in the ligand was able to show the different characteristics of ligands.

**TABLE 2:** Free energy contribution (kcal/mol) by residue and the corresponding free energy difference of H10.

Residue^a^	Dex-bound system	AZ938-bound system	Pred-bound system	Cor-bound system	DibC-bound system
L732	−1.54 (0.29)	−1.32 (0.20)	−1.11 (0.36)	−1.26 (0.25)	−1.30 (0.27)
L733	−0.18 (0.04)	−0.13 (0.04)	−0.12 (0.05)	−0.13 (0.04)	−0.12 (0.04)
N734	−0.06 (0.03)	−0.02 (0.02)	−0.05 (0.04)	−0.03 (0.03)	−0.03 (0.03)
Y735	−1.97 (0.37)	−0.98 (0.23)	−1.40 (0.61)	−1.41 (0.33)	−1.99 (0.52)
C736	−1.07 (0.32)	−1.06 (0.24)	−1.76 (0.63)	−1.06 (0.33)	−1.06 (0.23)
F737	−0.00 (0.03)	0.02 (0.02)	0.00 (0.04)	0.02 (0.02)	0.01 (0.02)
Q738	−0.00 (0.03)	0.01 (0.02)	0.03 (0.03)	−0.02 (0.03)	−0.02 (0.03)
T739	−2.35 (0.64)	−0.23 (0.13)	−0.93 (0.56)	−2.52 (0.52)	−2.65 (0.45)

a Numbers in the parentheses are the standard deviations.

### Elucidation of Allosteric Communication Pathway in Two Chosen Residues

**FIGURE 8:**
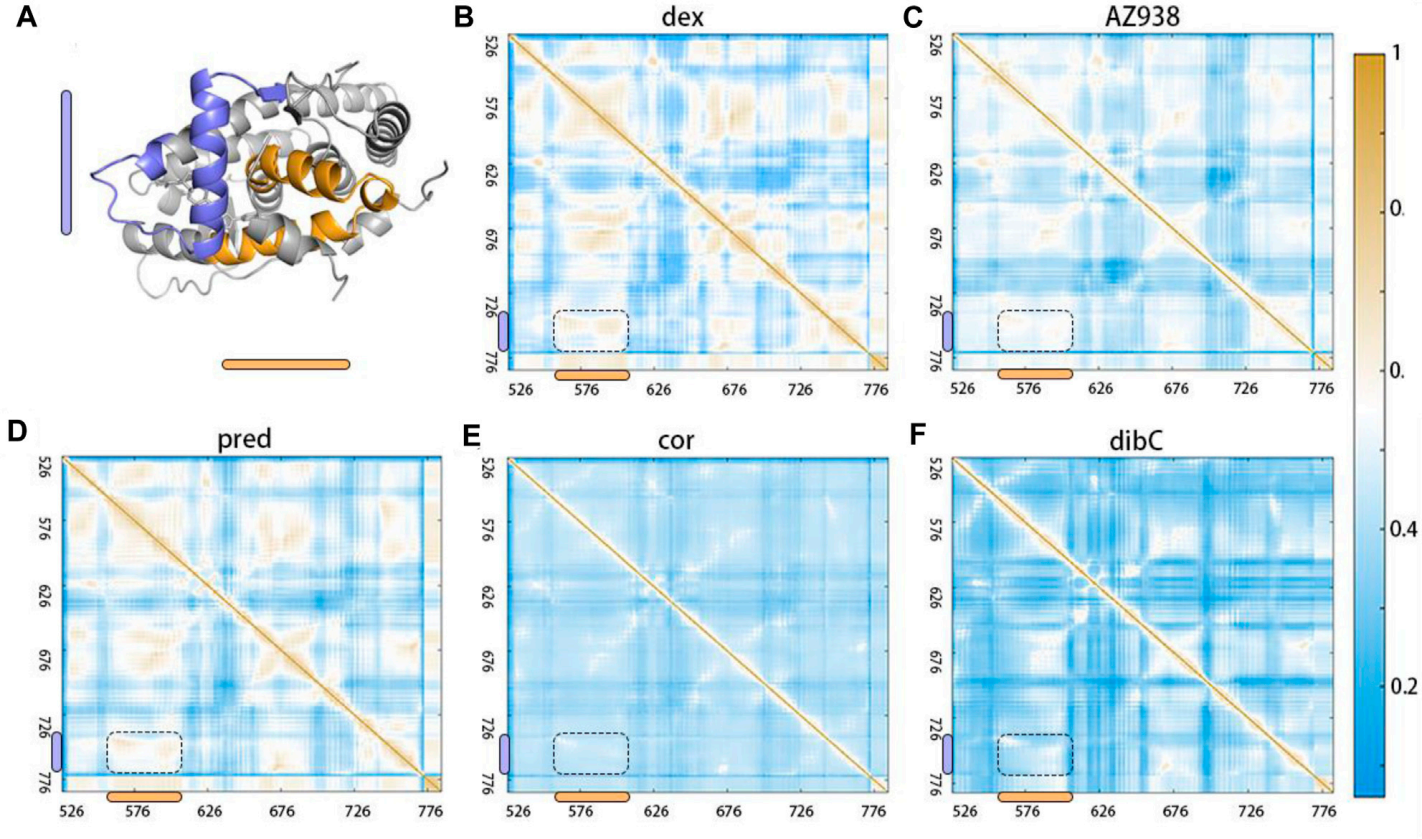
Dynamic cross-correlation matrix (DCCM) calculations with obvious differences in the region shown in **(A)**. DCCMs of **(B)** dex-bound system, **(C)** AZ938-bound system, **(D)** pred-bound system, **(E)** cor-bound system, and **(F)** dibC-bound system. Positive regions (yellow) stand for correlated motions, whereas negative regions (blue) represent anti-correlated motions.

After identifying the critical T739 and D590 in the ligand-binding pocket and cofactor-binding pocket, we next tried to explore the potential allosteric pathways connecting them. Using dynamic cross-correlation matrix (DCCM) calculations, we provided an overview of the inter-residue correlations within the simulation systems (Wang et al., 2022). Residues distributed in regions representing two sets of residues located near the ligand-binding pocket and cofactor-binding pocket demonstrated the biggest changes in the whole system. As shown in Figure 8, compared to the cor-bound system and dibC-bound system, the dex-bound system exhibited significantly increase correlated motions among distant residues. In the dex-bound system, obvious correlations between around G568 and around D590 suggested communication between H3 and H4–H5 (Figure 8B), indicating a certain correlation within the cofactor-binding pocket. Particularly, in the dex-bound system, the correlation of inter-molecular motions among the region near the ligand-binding pocket and cofactor-binding pocket colored by yellow and blue bars (framed using black dash lines) was compellingly strengthened than the other four systems. Pred-bond and AZ938-bound systems possessed weaker correlated motions in this region than dex-bound systems (Figures 8C, D) but were relatively stronger than the dibC-bound and cor-bound systems (Figures 8E, F). Weakened correlative movements between the ligand-binding pocket and the cofactor-binding pocket in dibC-bound and cor-bound systems suggested impaired signal propagation pathways between the ligand-binding pocket and cofactor-binding pocket. The degree of correlated motion levels in five systems could also partly reflect the different allosteric regulations among the five systems. Notably, the two residues discussed before were also in this region, which served as another evidence for their role in allosteric communication between the two pockets.

**FIGURE 9:**
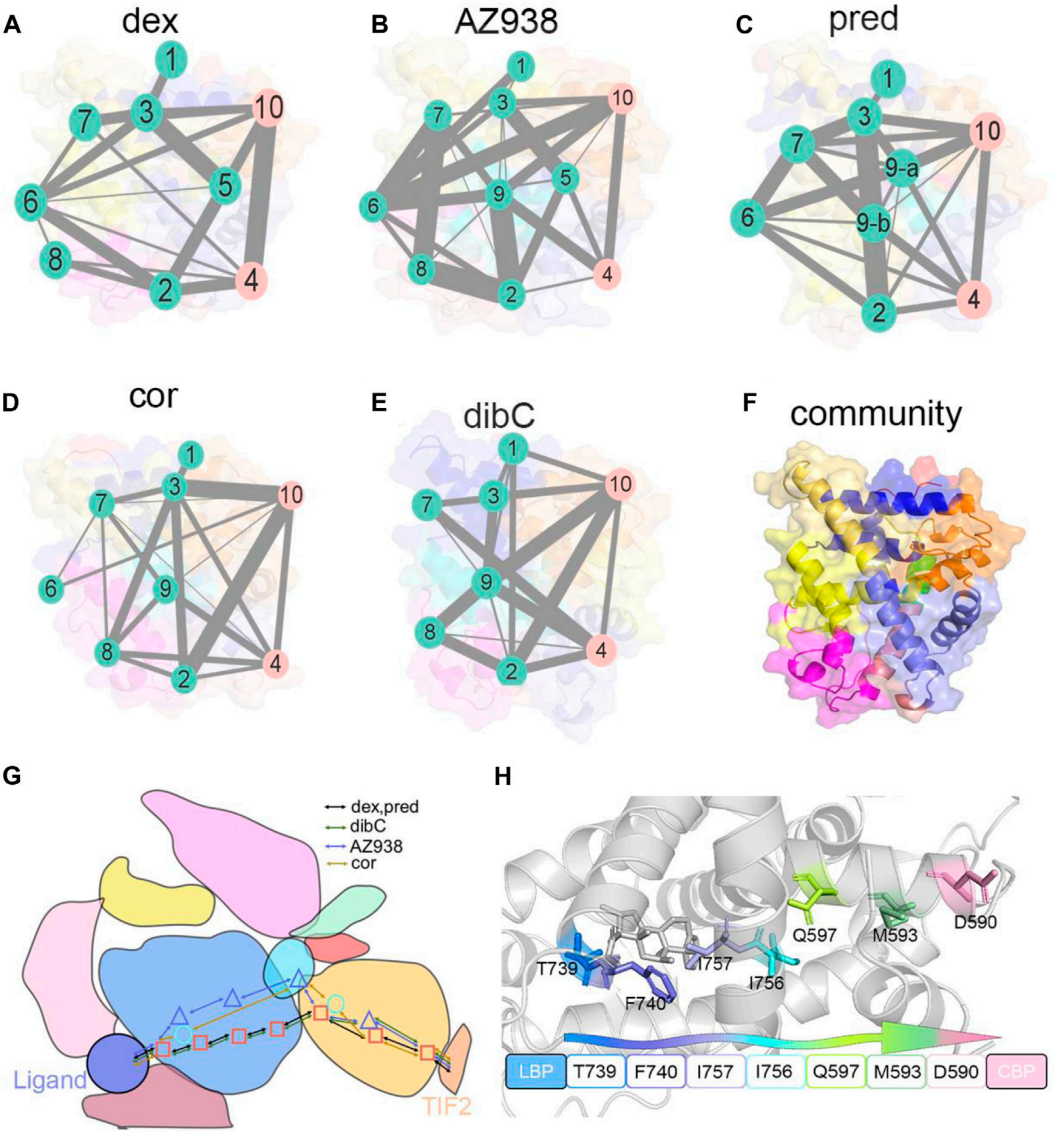
Community networks and the allosteric signaling pathways in each simulation system. Community network representation of **(A)** dex-bound system, **(B)** AZ938-bound system, **(C)** pred-bound system, **(D)** cor-bound system, and **(E)** dibC-bound system. **(F)** cartoon representation of cluster distribution in GR. Each sphere represents an individual community, and the thickness of sticks connecting communities is proportional to the corresponding edge connectivity. Schematic representation **(G)** of the domain-level allosteric signaling pathway and cartoon representation of signal propagations **(H)** connecting D590 and T739 in five systems.

Next, community network analysis and allosteric pathway analysis were carried out for the five systems to systematically investigate the allosteric networks (Wang et al., 2021). During the three replicas of simulations, residues that distanced within a 4.5 Å cut-off for at least 75% of the time were categorized into the same community, which could be seen as a congenerous unit within the systems (Qiu et al., 2021; Zhuang et al., 2022). As shown in Figures 9A–E, different systems were divided into different quantities of communities. Each community was represented by a colored circle and was superimposed on the 2D structure of the corresponding protein complex to reflect the relative positions with adjacent communities. Based on graph theory and topology, each community’s structural information flow was calculated (Sethi et al., 2009). The width of lines connecting two communities was proportional to the corresponding edge connectivity which was defined by the number of shortest paths passing through the edging nodes. In general, the residual components of each community were similar in the five systems. However, discrepancies between different systems still occurred. In the AZ938-bound system (Figure 9B) and dibC-bound system (Figure 9E), the complex was divided into 10 groups and eight groups, respectively, while in the other four systems, the complexes were divided into nine communities. Some communities were not consistently existed in all the five systems. For instance, community 9 was absent in the dex-bound system and community 6 was absent in the dibC-bound system. However, community 4 and community 10 consistently existed in five systems. They contained domains regarding the ligand-binding pocket and cofactor-binding pocket and the constituent residues within were similar among the five systems, indicating a critical role of these domains in allosteric communication. In the dex-bound system (Figure 9A), the connection between communities 4 and 10 was direct and strong. In contrast, the connection of communities 4 and 10 was much weaker in dibC- and cor-bound systems (Figures 9D, E), suggesting less informational communication through these two communities in these two systems. The thickness of the lines in communities 4 and 10 was in positive correlation with the order of efficacies of five systems, indicating that the communication between these two parts of the ligand-binding pocket and cofactor-binding pocket might dominate the differences in the ligand’s efficacy. However, the connection of communities 4 and 10 in dibC- and cor-bound systems were relatively weak, suggesting some structural impairment in these two systems. Such loosen connection in dibC-bound and cor-bound systems may due to the lack of community 5. In the dex-bound system, community 5 served as a major hub for information transduction. It connected communities 2 and 10, which indirectly strengthened the connection between communities 4 and 10. A similar impact was also observed in community 9 in AZ938-bound and pred-bound systems (Figures 9B, C). Notably, D590 and T739 were located at community 10 and community 4, respectively, suggesting that these two residues also participated in domains that drive the communication pathways in these two communities.

Additionally, by calculating the optimal and suboptimal pathways that link D590 in community 10 and T739 in community 4, we revealed the potential allosteric pathways between the chosen residues in the five systems. As shown in Table 3, the number of residues involved in the optimal pathways from T739 to D590 was similar in the five systems. However, the AZ938-bound system, pred-bound system, and dex-bound system displayed shorter optimal pathways, with a length of around 300, which indicated a stronger relationship between two chosen residues than in cor- and dibC-bound systems. (Figures 9G, H). Therefore, it could be concluded that the allosteric pathway between D590 and T739 was stronger in dex- and AZ938-bound systems than that in dibC- and cor-bound systems, which might also influence the efficacy of ligands. These results, together with DCCM analyses, collectively demonstrated that ligand-induced allosteric communications between the ligand-binding pocket and cofactor-binding pocket were one of the driving forces for the discrepancy of ligand’s efficacy.

**TABLE 3:** Allosteric pathway analysis between D590 and T739.

	Length	Residue	Pathway
Dex-bound system	309	7	590, 593, 597, 756, 757, 740, and 739
AZ938-bound system	272	7	590, 594, 597, 600, 733, 735, and 739
Pred-bound system	303	7	590, 593, 597, 756, 757, 740, and 739
Cor-bound system	362	6	590, 593, 596, 600, 736, and 739
DibC-bound system	451	7	590, 594, 597, 756, 757, 740, and 739

## Discussion

GR, as an essential nucleus receptor, controls a myriad of cellular functions and signal transduction (Fowden et al., 1998; Kumar and Thompson, 1999; Meijer et al., 2018; Liu B. et al., 2019). Upon the binding of ligands, GR is activated and induces conformational changes, involving post-translation modifications such as acetylation and phosphorylation. GR then translocates into the nucleus, where GR exerts its actions through transactivation and transrepression mechanisms (Vandevyver et al., 2014), regulating various metabolic functions. Thus, GR has been used to treat various metabolism and immunological disorder-related disease. Despite its broad clinical application, the serious side effects have always bothered patients and doctors. The underlying mechanisms of allosteric communications in GR may be an instructor in GR drug designs. Allosteric communication in the N-terminal domain and DNA-binding domain of GR has been detailly elaborated by Hilser and coworkers (Li et al., 2017). However, how ligands drive the allosteric effects and influence signal transductions remain unknown. Herein, by using MD simulations, we provided structural insights into the different allosteric effects induced by different ligands, thereby motivating progress in targeting GR’s ligand-binding domain for drug discovery.

By comparing the representative structures extracted from the three replicas of simulations, we revealed conformational dynamics in five systems bound to five different ligands (Figure 2). Conformational discrepancies in the ligand-binding pocket were largely due to the different chemical structures that ligands possessed, resulting in different degrees of openness in the ligand-binding region of H7 and H10. Conformational differences at the cofactor-binding pocket appeared much more magnificent. The directions of the TIF2’s conserved LXXLL helix in different systems strictly follow the order of ligand’s efficacy, with the dex-bound system having the closest distance between H4 and TIF2 and the dibC-bound system having the farthest one. This result was further testified by two pairwise distance measurements between D590 and the two ends of TIF2 (Figure 3). MM/PBSA analysis of residues near the cofactor-binding pocket and hydrogen bond analysis revealed that D590 on H4 was likely to be a potentially vital residue to have an impact on the conformation of TIF2.

Two-dimensional landscapes of two parameters relative to the ligand-binding pocket and cofactor-binding pocket separately were projected in five GR-ligand-TIF2 and five GR-ligand systems (Figure 4). The parameter representing the cofactor-binding pocket used the area of the triangle formed by three high MM/PBSA contribution residues. The parameter representing the cofactor-binding pocket used another three residues in the cofactor-biding pocket. By comparing landscapes from before and after the TIF2’s binding, changes occurred both in the ligand-binding pockets and cofactor-binding pockets in the five pairs of systems, suggesting the influences of allosteric communication between two pockets in all the systems. Representative structures in the five pairs of two-dimensional landscapes were aligned and compared. Various degrees of expansion occurred in H3, while evident expansion of H4 only occurred in the dex-bound system and AZ938-bound system, which might be related to the exceeding efficacy of these two systems (Figure 5). Distance between two atoms in D590 and R+2, respectively, that formed a hydrogen interaction was also analyzed (Figure 7). Dex-bound system appeared to be the most preferential one for the formation of the hydrogen interaction, while dibC-bound and cor-bound systems had an extra peak at distance beyond 3.5 Å, suggesting less preferential conformations for hydrogen interaction. This hydrogen bond was believed to be a crucial interaction between the TIF2 and GR. Thus, the different abilities of forming the hydrogen bond in these systems might influence the efficacy of ligands. MM/PBSA analysis and distance measurements were conducted on residues around the ligand-binding pocket, and T739 was identified as an important residue with large MM/PBSA contribution and hydrogen interaction with the ligand. Distance analysis of T739 and the ligand was able to show the different qualities of ligands’ binding in different systems. By applying DCCM, inter-residue correlations were investigated among the five ligands (Figure 8). A distinguishable discrepancy was found in correlations of the region relative to the ligand-binding pocket and cofactor-binding pocket. In the dex-bound system, the correlation was the strongest, while in dibC-bound and cor-bound systems, the correlation was much weaker, suggesting impaired allosteric communication in the two complexes. Notably, D590 and T739 were also in this region, implying their participation in the allosteric communication. To systematically investigate the allosteric networks, community network analysis and allosteric pathway analysis were carried out (Figure 9). We observed different levels of communication between group 4 and group 10, which was consistent with the ligands’ efficacy (Figure 9). In addition, from community analyses and suboptimal pathway analysis, we found that the allosteric propagation pathway between two representative residues in the ligand-binding pocket and cofactor-binding pocket in five systems.

In view of the crucial role played by GR in clinical treatments (Van Staa et al., 2000), the development of new drug targeting GR has been the major focus over the past few decades. Thitherto, few accomplished design drugs with high efficacy and low side effects. This is largely due to the obstacles in the lack of knowledge of GR’s allosteric effects (Ni et al., 2021). The underlying mechanisms of what induces the discrepancy in agonists’ efficacy remain elusive. Thus, our study focusing on the allosteric communications of GR’s conformational dynamics is useful. Moreover, members of the NR family possess mutual structures with similar sequences. The TIF2 is the common cofactor that interacts with the AF2 interface of NRs. Thereby, it is presumable that the mechanism we unveiled in the GR also applies to others in the NR family and therefore has a more generalized value. Taken together, our study elucidated the driving force behind the ligands’ efficacy induced by different agonists’ binding as well as the detailed mechanism of allosteric communication between the ligand-binding pocket and cofactor-binding pocket. Our explorations of the conformational outcomes induced by the binding of different ligands have provided insights for new drug design by conditional genome manipulation or modifying ligand’s interactions with its pocket.

## Data Availability

The original contributions presented in the study are included in the article/Supplementary Material; further inquiries can be directed to the corresponding authors.
