# Supplementary material for: The Role of Conformational Dynamics and Allostery in the Control of Distinct Efficacies of Agonists to the Glucocorticoid Receptor
Source: Front Mol Biosci. 2022 Jul 7;9:933676. doi: 10.3389/fmolb.2022.933676 (PMC9300934; doi:10.3389/fmolb.2022.933676)
Supplement: Supplementary file 1 [file Table1.DOCX]

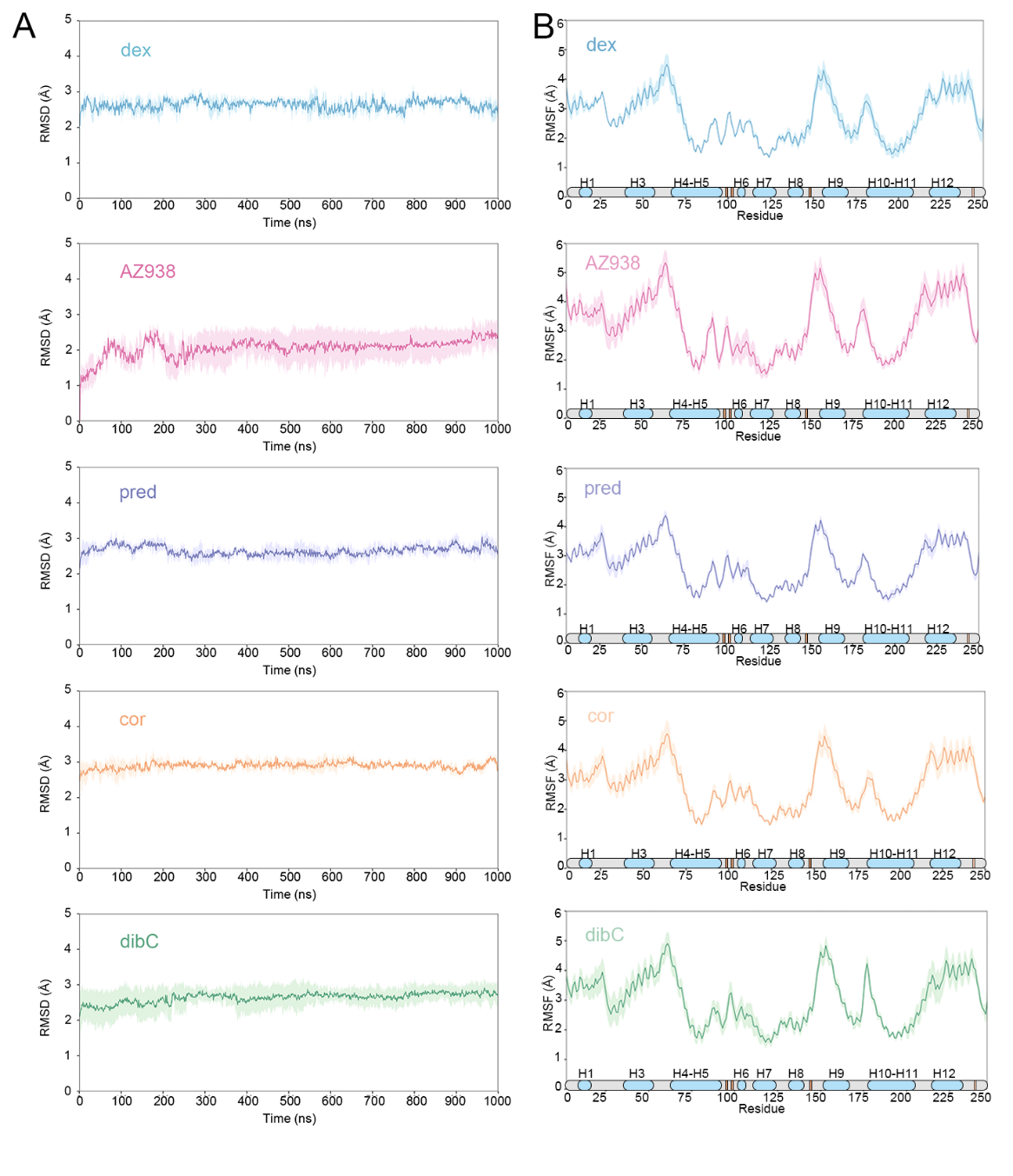


**Figure S1:** (A) Time evolution of the root-mean-square deviation (RMSD) of the glucocorticoid receptor backbone atoms with respect to the initial structure in five systems with different agonists. The average of three replicas (dark line) is indicated with standard deviation (thin, translucent, vertical line). (B) Root-mean-square fluctuations (RMSF) of glucocorticoid receptor atoms averaged over three production runs with standard deviations indicated as a thin, translucent, vertical line. The horizontal axis signifies the domain organization of glucocorticoid receptor.
